# Fuzzy Evaluation of Glycemic Load and Beta-Glucan Intake: Deriving a Dietary Circle Oriented Toward Type 2 Diabetes Prevention

**DOI:** 10.3390/nu18213666

**Published:** 2026-11-04

**Authors:** Jan Wirsam, Bernd Wirsam, Heike Schick, Sabine Poschwatta-Rupp

**Affiliations:** 1 Campus Treskowallee, HTW Berlin, 10313 Berlin, Germany; 2 Independent Researcher, Schubertstr. 4, 35440 Linden, Germany; 3 GOE mbH, Am Gemeindeplatz, 18375 Prerow, Germany; 4 Ernährungskonzepte, Gothaer Straße 17, 35396 Gießen-Wieseck, Germany

**Keywords:** type 2 diabetes, glycemic load, beta-glucan, fuzzy sets, food-based dietary guidelines, dietary optimization

## Abstract

**Background/Objectives:** Diabetes mellitus type 2 (T2D) is a global epidemic affecting over 500 million adults. Glycemic load (GL) and beta-glucan intake are modifiable dietary factors related to T2D risk that are largely shaped by the choice of cereal-based foods. This theoretical study derives fuzzy sets for GL and beta-glucan to enable their joint nutritional evaluation within a multidimensional framework and uses the framework to derive a dietary circle oriented toward T2D prevention. **Methods:** Fuzzy membership functions for GL and beta-glucan were constructed based on epidemiological dose–response data, guideline values, and authorized health claims. These functions were integrated into an existing fuzzy evaluation system that aggregates the evaluations of nutrient intake using a Schweizer–Sklar T-norm (*p* = 6). A fuzzy optimization algorithm was applied to the food quantities underlying the current German food-based dietary guidelines. **Results:** The GL fuzzy set evaluates intakes below 80 g/d as desirable and penalizes intakes above 160 g/d; the beta-glucan fuzzy set evaluates intakes of at least 3 g/d as good, with an optimum at 6 g/d. Fuzzy optimization shifted the diet markedly toward vegetables, legumes, fish, and tofu while strongly reducing cereals and milk products, and GL decreased from 161 to 77 g/d. The resulting dietary circle achieved an evaluation > 0.9 for the intake of every nutrient considered and an aggregate score of 0.894. **Conclusions:** Integrating GL and beta-glucan into the fuzzy evaluation framework yields a coherent, food-based dietary circle oriented toward T2D prevention that can serve as a reference for counseling, menu planning, and intervention studies; its preventive effect remains to be tested.

## 1. Introduction

Worldwide, more than 500 million adults are living with diabetes [1], with T2D being the most common form.

According to the International Diabetes Federation, 537 million adults (10.5%) were living with diabetes in 2021, a number projected to rise to 783 million by 2045; diabetes and its complications—cardiovascular disease, kidney failure, retinopathy, neuropathy, and amputations—caused 6.7 million deaths and health expenditures of 966 billion USD in 2021 [1]. The highest prevalence is found in Pakistan (30.8%), in Pacific Island states, and in Gulf states such as Kuwait (24.9%), and the largest absolute numbers in China and India; in general, prevalence is higher in urban than in rural areas and in high-income than in low-income countries [1]. In contrast, populations with traditional or plant-based lifestyles show strikingly favorable metabolic profiles: the Tsimane in the Bolivian Amazon, who eat a diet rich in unprocessed, fiber-rich carbohydrates and are physically very active, have low blood glucose levels [2], and among Seventh-day Adventists in North America, T2D prevalence was 2.9% in vegans compared with 7.6% in non-vegetarians [3]. Because the increase in T2D is driven largely by modifiable lifestyle factors, diet is a key starting point for prevention.

A large body of evidence documents a strong association between various dietary patterns and the subsequent incidence of T2D, including the influence of a low glycemic index, intake of beta-glucan, and various plant-based diets [4]. The DDG (German Diabetes Society) [5] primarily recommends adherence to DGE (German Society of Nutrition) guidelines [6], in particular the preference for complex carbohydrates and whole grain products, due to the slower rise in blood glucose levels they produce. Further recommendations include limiting sugar intake to a maximum of 50 g/day, a plant-based and fiber-rich diet (vegetables and fruit, 5 a day) due to high nutrient density and reduction in the risk of secondary diseases. Legumes and vegetable oils are recommended, as is reducing the consumption of red meat and processed meat products (max. 300 g per week) and low-fat fish. Water or unsweetened tea should be the preferred beverages, while sugar-sweetened drinks should be avoided. Physical activity directly after meals is also encouraged [5].

Because the diet resulting from these recommendations depends heavily on the type of diabetes, body weight, meal timing, and medication, individualized dietary and lifestyle counseling by certified professionals is always recommended [5].

Large studies with over 25,000 participants were analyzed using the latest pattern recognition algorithms, clustering methods and scoring systems [7], with respect to both genetic risk groups (genetic risk score, GRS) and dietary risk groups (diet risk score, DRS). Participants had no T2D at the start of the study. The occurrence of T2D was monitored in follow-up examinations over a period of 17 years. Both genetic and dietary risk factors were found to contribute to risk independently and additively. A particularly elevated T2D incidence was associated with higher intake of processed meat and sugar-sweetened beverages, whereas a reduced risk was observed with higher intake of whole-grain products and coffee [7].

Smith et al. [8] performed the most comprehensive analysis to date, identifying genetic clusters related to T2D in 1.4 million people, though without reference to nutrition. Membership in each cluster is represented by a score. Some clusters are strongly associated with nutrition; for example, the cholesterol cluster is dominated by the APO-E polymorphism.

A meta-analysis of prospective cohort studies on the relationship between GL and T2D risk [9] found that GL is robustly associated with increasing T2D incidence. The relative risk is much greater than the risk associated with insufficient fiber or whole grain intake.

GL and beta-glucan are, however, not independent targets for dietary optimization: both are shaped primarily by the choice of cereal-based foods. Oats and barley are the main dietary sources of beta-glucan.

The aim of this theoretical study was therefore (i) to derive fuzzy sets for GL and beta-glucan oriented toward the prevention of T2D, (ii) to integrate them into an existing fuzzy evaluation framework together with the fuzzy sets for all other nutrients, and (iii) to apply fuzzy optimization to the food quantities underlying the current German food-based dietary guidelines in order to derive a dietary circle oriented toward T2D prevention. This dietary circle is intended as a directional target for primary prevention, not as a recommendation for the dietary management of manifest T2D.

## 2. Materials and Methods

### 2.1. Fuzzy Evaluation of Nutrient Intake

The evaluation of nutrient intake using fuzzy sets and the algorithm for dietary optimization were described as early as 1997 [10], applied for ten years in the DGE standard program [11], and are still in use in the OptiDiet software(Rev 6.0) [12], particularly for personalized dietary counseling for individuals with specific requirements (athletes, special diets, allergies).

In the standard fuzzy sets, intakes that comply with the DGE recommendations are evaluated with values above 0.9, and deviations from them with values below 0.9.

Each fuzzy set is defined by up to five support points: the evaluation at zero intake, the lower limit of the desirable range (“lower good”, evaluation 0.9), the optimum (evaluation 1.0), the upper limit of the desirable range (“upper good”, evaluation 0.9), and the intake above which the evaluation is clearly poor (“upper bad”, evaluation 0.1). Adjacent support points are connected by cubic spline functions, so that the membership function is smooth and continuously differentiable. Above the “upper bad” point, the evaluation decreases exponentially, so that a direction of improvement remains defined even for unrealistically high intakes. By definition, an evaluation of at least 0.9 corresponds to compliance with the respective recommendation [6,10]. The figures in this article show the membership functions over the intake range typical of realistic dietary records.

In this study, suitable fuzzy sets for GL and beta-glucan are derived with a focus on the prevention of T2D. Together with the complete set of fuzzy sets for all other nutrients, a dietary circle can then be calculated. Besides personalized nutrition counseling, the optimization is also suited to calculating menu plans for residential institutions and for intervention studies.

### 2.2. Aggregation of Nutrient Evaluations

Focusing on GL and beta-glucan alone leads to conflicting objectives, especially with respect to saturated fatty acids and dietary cholesterol. Therefore, it is necessary to apply aggregation with the fuzzy sets of all other nutrients.

Overall dietary evaluation is generally based on 7-day dietary records. In addition to evaluating individual nutrient intakes, an overall evaluation is needed to weigh improvements in one nutrient against deteriorations in another, and to define an optimizable objective function. The overall evaluation is performed using an aggregation operator, in our case the Schweizer-3 operator [13], which is defined as shown in Equation (1):

T(x,y) = 1 − [(1 − x)^p^ + (1 − y)^p^ − (1 − x)^p^ ∗ (1 − y)^p^]^1/p^   p > 0 (1)

where x and y ∈ [0, 1] denote the fuzzy evaluations of the intakes of two nutrients, T(x,y) is their aggregated evaluation, and p > 0 is the parameter of the operator. For p = 1, the operator equals the algebraic product x·y; with increasing p, it approaches the minimum operator, so that the poorest single evaluation increasingly determines the overall result. Because the operator is commutative and associative, the evaluations of all *n* nutrients considered can be aggregated successively into a single overall evaluation [14].

The value *p* = 6 for the parameter *p* has proven effective in dietary counseling practice; the rationale for this choice is detailed in [14].

### 2.3. Optimization in a High-Dimensional Decision Space

One-dimensional view: As shown in [14], the influence of a single foodstuff on the overall intake evaluation can be illustrated graphically. A typical one-dimensional decision is, for example, “to improve the intake of beta-glucan, consume more oats”.

Two-dimensional view: The interaction of two foodstuffs can also still be illustrated graphically, as shown in two examples in [14]. A well-known example of two-dimensional decision-making in nutrition education is the “substitution tables” of the DGE, e.g., “Instead of bread at breakfast, prefer oatmeal” or “Instead of meat, consume more legumes as a source of protein”.

Three-dimensional and higher-dimensional view: Such interrelations can no longer be visualized graphically, and human intuition cannot grasp them. An *n*-dimensional fuzzy optimization algorithm or an AI can handle them very well by following the gradient of the objective function to approximate a solution. The procedure is therefore not intuitively comprehensible to a nutritionist who thinks in one or two dimensions.

This also applies to many decision problems outside nutrition. Most of them are not crisp systems and occur in high-dimensional decision spaces [15]. The approximate solutions calculated in this way are usually not intuitively understandable to human thinking and are, in this sense, superior to intuitive solutions.

### 2.4. Food Composition Data and Optimization Procedure

All calculations were performed with the OptiDiet software (Version 6.0) [12]. Nutrient intakes were calculated from the German food composition database BLS 3.02 [16]. Because the BLS contains neither GL nor beta-glucan, these values were added for the food groups required, using GL values from the international tables of glycemic index and glycemic load [17] and beta-glucan contents from [18]. For this purpose, the OptiDiet function for creating new food entries was used: an original BLS-coded food was copied, and the GL and beta-glucan contents were added to the copy. All calculations were therefore performed with copies of the original BLS entries supplemented in this way.

In the optimization, the decision variables are the intake quantities of the food groups, which are constrained to non-negative values; the objective function is the aggregated fuzzy evaluation of all nutrients considered according to Equation (1). Starting from a given diet, the algorithm changes the food quantities iteratively in the direction in which the objective function improves. The OptiDiet implementation is proprietary; it combines gradient-based search steps with elements of genetic algorithms.

## 3. Results

### 3.1. Determination of Fuzzy Sets for GL and Beta-Glucan

To determine a fuzzy set for GL for diabetes prevention, the dose–response meta-analyses of prospective cohort studies by Livesey et al. [19,20] were used as benchmark data. These studies found that a diet with a high GL constitutes a substantial increase in T2D risk, independent of fiber intake. Across the range of typical intakes (approximately the 10th to 90th percentile), a glycemic index (GI) of 76 was associated with an 87% higher risk of developing T2D than a GI of 48. For GL, which Livesey et al. report directly in g/d standardized to a 2000 kcal diet, the corresponding range from 73 to 257 g/d was associated with an 89% higher risk, and each increment of 80 g/d with a 26% higher risk [19].

The DDG [21] recommends a GL of <10 g per serving or meal as a guideline value; a value of 11–19 g is considered “moderate”, and a value of ≥20 g “concerningly high”. To scale these serving-based values to daily intake for a 2000 kcal/d diet, a factor of 8 was used, corresponding to approximately eight servings or eating occasions per day. The recommended value is thus <80 g/d; values of 80–160 g/d are rated as “moderate”, and values > 160 g/d as “concerningly high”. This extrapolation is consistent with the epidemiological data: the lower threshold of 80 g/d is close to the lowest typical intake of 73 g/d in [19], and the width of the moderate range corresponds to the GL increment of 80 g/d that was associated with a 26% higher T2D risk [19].

This results in the fuzzy set shown in Figure 1: intakes up to 80 g/d are evaluated with at least 0.9, and the evaluation decreases continuously at higher intakes.

For energy requirements deviating from 2000 kcal/d, the support points are scaled accordingly; the reference is the energy requirement (here, the weighted mean energy requirement of 1930 kcal/d; Section 3.2), not the actual energy intake. This fuzzy set aligns with DDG recommendations and is consistent with the findings from the dose–response meta-analyses [19,20]. It is primarily intended to reduce the risk of developing T2D and is therefore initially designed for prevention.

Analogously, a fuzzy set for beta-glucan was derived (Figure 2). Its lower support point is based on the authorized health claim that a daily intake of at least 3 g of beta-glucan from oats or barley contributes to lowering blood LDL cholesterol [22]; a randomized placebo-controlled trial confirmed the effect of 3 g/day on fasting lipids and glucose as well as its good tolerability [23]. Accordingly, the membership function reaches 0.9 at 3 g/day. Effects on postprandial glycemia, which are more directly relevant to T2D prevention, require higher amounts: the authorized claim on the reduction in postprandial glycemic responses refers to 4 g of beta-glucan per 30 g of available carbohydrate per meal [22] (see [24] for the recent modification of this claim), and a meta-analysis of controlled feeding trials found a linear dose–response relationship, with each additional gram of oat beta-glucan per 30 g of available carbohydrate reducing the postprandial glucose response by about 8% [25]. The optimum was therefore not placed at the minimum effective intake but at twice this amount (6 g/day). This also reflects a general principle of the fuzzy approach: in an imprecise system such as nutrition, the optimum should not lie on the boundary of the acceptable range. The upper support point (0.9 at 12 g/day) is based on the authors’ judgment that higher intakes can hardly be achieved with conventional foods and may increasingly impair gastrointestinal comfort. Because excess beta-glucan is not toxic, the membership function does not fall to zero at high intakes but declines gradually. Thus, the evidence for cholesterol lowering is well established at 3 g/day, the evidence for glycemic effects relates to higher doses, and direct evidence for the prevention of T2D is still lacking; the beta-glucan fuzzy set should be interpreted accordingly.

### 3.2. Derivation of a Food-Based Dietary Circle Oriented Toward Diabetes Prevention

For the revision of the food-based dietary guidelines (FBDG), the DGE developed a new methodology [26]. Using data from the German National Nutrition Survey II (NVS II) [27], conducted in 2005–2006, the food consumption of a representative sample of the German population was recorded for a total of 15,371 subjects aged 14–80 years, stratified by age, sex, region, and season. It was observed that the population did not eat enough food of plant origin and consumed too much meat and meat products; this applied especially to men, who also drank too much beer [27].

The aim of that study [26] was to meet the DGE recommendations, based on the German food composition database (BLS 3.02) [16], via linear optimization along the shortest path. Ten kitchen units/day were required to achieve the optimization target. In addition, the reduction in disease burden and environmental impact was incorporated into the optimization by weighting [26]. The quantities thus determined were > 0 for 13 food groups, so that the “food space” comprises 13 independent food groups and therefore 13 dimensions. These groups can be represented as a dietary circle. The objective function was the weighted mean of the deviation from the dietary behavior observed in the NVS II, disease burden, and environmental impact. The result therefore does not represent the health optimum alone, neither for a population nor for an individual.

To describe the health optimum as a directional goal for reducing T2D risk, the following approach was taken:

The starting point was the “optimum” described in Table 3 of the DGE publication [28]. To ensure comparability with [26], vitamin D, fluoride, copper, and manganese were not included in our calculations; in the derivation of the DGE guidelines, they had also been excluded owing to data limitations (Table e1 in [28]). The recommendation to use iodized salt was taken into account by including enough iodized salt to reach an iodine intake of 177 µg/d, close to the recommendation of 180 µg/d. As expected, the fuzzy evaluation yielded values above 0.9 for all other nutrients, indicating compliance with the recommendations. Energy intake of this starting diet was 2030 kcal/d, almost identical to the 2029 kcal/d given in [26], which corresponds to the mean energy intake observed in the original NVS II study. As the reference for the fuzzy evaluation, however, we used the weighted mean energy requirement of 1930 kcal/d according to the DGE reference values [6]. For all other nutrients, the goals were calculated by averaging the recommendations for women and men; only for alcohol and iron were the values for women used. These goals were also used in the fuzzy approach. Nutrient analysis was based on BLS 3.02 [16], extended by GL and beta-glucan (Section 2.4).

For GL, an intake of 161 g/d was calculated for this starting diet based on GL data for foods [17] and evaluated at 0.53 using the GL fuzzy set. This corresponds to a high intake, just above the value of 160 g/d at which the DDG classifies GL as “concerningly high”. For beta-glucan, an intake of 4.56 g/d was calculated based on beta-glucan data for foods [18] and evaluated at 0.99 (verbal interpretation: “optimal”), derived almost exclusively from the cereal food group, in which oats and barley were not considered separately.

Further steps were then taken based on the given food groups and intake quantities, moving toward the health optimum for T2D prevention while taking into account the evaluation of GL and beta-glucan intake. After four optimization steps, GL was reduced to 99.4 g/d; however, owing to irresolvable conflicts among the objective criteria, the overall evaluation reached only 0.81. This was due to excessive dietary cholesterol (315 mg/d) and an excessive intake of saturated fatty acids (12 E%). Thus, within the food groups of the DGE dietary circle, the GL target could not be reached without violating other nutrient criteria. To bring the results into a range that meets all criteria of the objective function, further food groups capable of resolving these conflicts had to be made available. Because cereals differ in their beta-glucan content, oatmeal and barley were separated from the other cereals, and because vegetables vary considerably in their carbohydrate content, the seven BLS subgroups (G1–G7) were used in the optimization instead of the main group (G = vegetables).

The search functions of OptiDiet were used to screen the BLS for food groups that are low in GL, yet provide energy, and contain neither dietary cholesterol nor saturated fatty acids in any significant quantity. Of the ten additional food groups offered to the optimization algorithm, only tofu and unsweetened, minimally processed soy products ultimately remained. To make the effects of beta-glucan visible, oats and barley were also included as a subgroup within cereals.

With the inclusion of the food group “Tofu and Soy Products” and the splitting of the main group vegetables (G) into the seven subgroups (G1–G7), the “food space” now reached 21 dimensions (including drinking water and unsweetened coffee and tea).

The optimization algorithm was then run again with the same goals as in [26]: to reach the recommendations and to stay as close as possible to the “optimum” calculated there. Our results are therefore not the optimum but a step in the right direction. As a result, the intake quantities of vegetables and legumes increased substantially once more, as did the intakes of fish, meat, nuts, and seeds. Tofu and soy products entered the diet in remarkable amounts. The intakes of potatoes, juices, vegetable oils, eggs, and sausage remained nearly unchanged, whereas the intakes of cereals, milk and dairy products, and butter and margarine decreased substantially. Without beverages, which are not shown in Table 1 or in the dietary circle, and with the subgroups combined into their main groups, the “food space” shown in Table 1 comprises 14 dimensions.

Table 1 shows the initial situation and the results of the optimization, including the evaluation of GL and beta-glucan. The algorithm achieved an evaluation of at least 0.9 for all nutrients considered, including GL (intake: 77.0 g/d, evaluation 0.91) and beta-glucan (intake: 3.2 g/d, evaluation 0.92). The contribution of the food groups to the intake of GL and beta-glucan is shown in the last column. The largest GL contributions came from cereals, bread, pasta, and rice (32.1 g/d), followed by vegetables and fruits (27.6 g/d, dominated by fruits); milk and dairy products (4.3 g/d), juices (3.8 g/d), potatoes (3.6 g/d), legumes (3.2 g/d), and nuts and seeds (2.0 g/d) contributed considerably less, and all other food groups were negligible. The contributions add up to 76.9 g/d; the difference from the total of 77.0 g/d is due to rounding. The main source of beta-glucan was cereals (3.1 g/d), of which barley contributed 2.4 g/d.

Energy intake decreased from 2030 kcal/d in the starting diet to 1630 kcal/d after optimization (Table 2). To distinguish the effect of the changed food composition from that of the lower energy intake, GL was also expressed per 1000 kcal: it decreased from 79.3 g/1000 kcal in the starting diet to 47.2 g/1000 kcal after optimization, i.e., by 40%. At equal energy intake, the GL of the optimized diet would thus be about 40% lower than that of the starting diet (about 96 g/d instead of 161 g/d at 2030 kcal/d). The reduction in GL therefore results predominantly from the changed food composition and only partly from the lower energy intake.

Saturated fatty acid intake was 9.6 E%, well within the target range, and dietary cholesterol at 106 mg/d was clearly below the threshold of 300 mg/d. The energy ratio of fat: carbohydrates: protein was 34:40:20 E%.

Evaluations ≥ 0.9 indicate intakes within the desirable range of the respective fuzzy set, which does not necessarily coincide with reaching the averaged recommendation. For vitamin B12, for example, the fuzzy set reaches 0.9 at 1.5 µg/d and its optimum at 4 µg/d, so that an intake of 2.9 µg/d is evaluated at 0.99.

The quantities in the third column of Table 1 serve as the basis for the dietary circle shown in Figure 3.

## 4. Discussion

The segment sizes depicted in the DGE Food Circle (Figure 4) cannot be directly derived from the weight-based quantities of the underlying optimization model [26] (Table e2 in [28], Scenario 1 Variant 3). The segment areas reflect portion sizes and frequencies (Table e3 in [28]) rather than proportional food weights, resulting in a visually different emphasis. The authors note that the translation into the DGE Food Circle used the existing German food-based dietary guidelines (FBDG) as a starting point rather than deriving the visual representation directly from the optimization quantities, yet the resulting divergence in proportional emphasis is not explicitly quantified.

The correct interpretation of such dietary circles also requires some comments. Vegetables and fruits have a high water content compared with some other food groups; therefore, their segments appear very large when segment sizes are based on weight. Another question is how much the evaluation deteriorates when intakes deviate from the calculated values. It was already established in [10] that, near the optimum in the high-dimensional “food space”, the evaluation field represents a stable and error-tolerant situation with respect to changes in food intake quantities. In [14], we demonstrated the influence of individual food items on the overall evaluation at the optimum. The situation here is different: the optimization was stopped as soon as the recommendations for all nutrients considered were met (evaluation > 0.9), because further optimization steps toward the optimum lead to changes far from common eating behavior. This means, however, that small changes in the wrong direction can clearly deteriorate the result. A study is currently being designed to test whether such an optimized diet—incorporating the evaluations of GL and beta-glucan within an otherwise fully optimized nutrient intake and moderate physical activity—leads to genotype-specific responses in relevant clinical traits.

The direction of the dietary changes produced by the optimization can be compared with epidemiological evidence on food groups and T2D risk. In a meta-analysis of prospective studies, higher intakes of whole grains, vegetables, fruits, and dairy products were associated with a lower risk of T2D, whereas red and processed meat and sugar-sweetened beverages were associated with a higher risk; for refined grains, legumes, nuts, eggs, and fish, no significant linear associations were found [30]. Higher soy intake has been associated with a 17% lower risk of T2D [31], and the prevalence of T2D among vegetarian Seventh-day Adventists is markedly lower than among non-vegetarians [3]. The increases in vegetables, fruits, and soy products in the optimized diet, as well as the low intake of meat and sausage (well below the DGE maximum of 300 g/week), are consistent with this evidence. Other changes are not: the overall reduction in cereals also affects whole grains, which are inversely associated with T2D, and the marked reduction in milk and dairy products contrasts with their inverse association with T2D risk. These reductions result from the high contribution of cereal products to GL and from the constraints on saturated fatty acids and dietary cholesterol, respectively, while oats and barley, the main sources of beta-glucan, were retained. Likewise, the increases in legumes and fish are not explained by direct associations with T2D, but by their contribution to the supply of protein and micronutrients at a low GL.

Such comparisons, however, have a fundamental limitation. Epidemiological associations describe one-dimensional dose–response relationships between single food groups and disease risk, adjusted for other factors. The optimization, in contrast, operates in a high-dimensional decision space in which each food group is evaluated by its combined effect on all nutrients simultaneously (Section 2.3). A food group may therefore increase or decrease in the optimized diet not because of its own association with T2D but because it resolves conflicts between other objectives, as shown here for tofu and soy products, which provide protein and energy at a low GL without adding saturated fatty acids or cholesterol. The result of a high-dimensional optimization can therefore not be validated by one-dimensional dose–response relationships alone; this requires intervention studies with the complete diet.

Compared with established dietary patterns, the optimized diet shares essential features with the Mediterranean diet, which reduced the incidence of T2D by 52% in the PREDIMED-Reus trial [32]: high intakes of vegetables, fruits, legumes, nuts, and fish, and low intakes of meat and butter. It differs from the Mediterranean diet in its lower proportion of easily digestible carbohydrates, the absence of alcohol, the inclusion of soy products, and the comparatively small quantity of vegetable oils. Traditional diets associated with a very low risk of metabolic disease, such as that of the Tsimane, are rich in carbohydrates (about 72% of energy), but these come mainly from unprocessed, fiber-rich foods and are combined with a very high level of physical activity [2]. These examples show that a low risk of T2D is compatible with different food patterns. The approach presented here goes beyond descriptive dietary patterns in that it derives quantitative intakes that simultaneously meet all nutrient recommendations considered and the criteria for GL and beta-glucan.

Finally, the fuzzy evaluation differs fundamentally from conventional, non-fuzzy approaches. Conventional nutrient calculation programs compare intakes with reference values and report deviations as percentages, usually with crisp thresholds: an intake of 99% of a recommendation is classified as insufficient, whereas an intake of 300% is classified as adequate, although it may be less desirable. Moreover, percentage deviations of different nutrients cannot be combined into a meaningful overall evaluation, so that no objective function for an optimization is available. Linear optimization, as used in [26], replaces this by crisp constraints; its solutions characteristically lie on the boundaries of the feasible region, i.e., exactly at the minimum or maximum values of individual recommendations. Because official recommendations are intended for population groups and food composition data are imprecise, such boundary solutions are fragile. The fuzzy approach evaluates every intake on a continuous scale, including excessive intakes, and aggregates all evaluations into one objective function whose optimum lies within the range of acceptable intakes [10,14]. A formal quantitative comparison of fuzzy and non-fuzzy evaluation of the same diet was beyond the scope of this study.

Like the DGE dietary circle, the dietary circle presented here represents one of several feasible solutions—an idealized directional target on the path toward a health-promoting dietary pattern oriented toward T2D prevention. It is not intended as a recommendation for the dietary management of manifest T2D, which—particularly in insulin-treated patients—requires individual coordination of the amount and timing of food intake, insulin dosage, and physical activity [5]. In such cases, both the current blood glucose level and its trend are decisive for the amount and timing of food intake as well as for the timing and dose of insulin administration.

The environmental impact aspects incorporated in [26] correlate with the fuzzy evaluation, as demonstrated in [33]. They can be included in the OptiDiet algorithm but need not be.

## 5. Limitations

The present study has several limitations that should be acknowledged.

Dependence on reference values. The fuzzy membership functions—and therefore the entire evaluation field—are based on the DGE reference values [6]. Any revision of these values directly alters the membership functions and shifts the optimum.

Nature and sources of the evaluation criteria. The fuzzy evaluation is not an additive scoring system in which points are summed: each membership function evaluates the intake of a single nutrient, and the evaluations are combined by a T-norm, so that a poor evaluation of one nutrient cannot be compensated by very good evaluations of others [14]. The criteria for GL (DDG classification, cohort data) and beta-glucan (health claims, intervention trials) necessarily come from different sources, and the upper support point for beta-glucan is partly based on expert judgment. Shifting the support points would change the evaluations and possibly the optimized food quantities. Sensitivity analyses with alternative support points and aggregation parameters were not performed in this study; the choice of p = 6 has been calibrated and documented in [14].

Population specificity. The reference values, the starting diet, and the food composition data refer to the German population (NVS II, BLS). Genetic clusters of T2D have been characterized across ancestries without reference to nutrition [8], and dietary and genetic risk scores have been combined in a Swedish cohort [7]. Transferring the present results to populations with other dietary habits, food supplies, or genetic backgrounds requires further research, and analyses based on data from other populations may yield different results.

Inherent imprecision of nutritional data. Nutrition is an inherently imprecise system: nutrient contents vary with variety, growing conditions, and preparation; food composition databases introduce measurement uncertainty; and many official reference values are estimates. The fuzzy approach was chosen partly in response to these limitations but does not eliminate them.

Theoretical nature. This study presents and illustrates a theoretical mathematical framework. Model outputs are computed purely from nutrient content data and do not take into account digestibility or nutrient–nutrient interactions. No preventive effect of the derived dietary circle has been tested; this requires intervention studies such as the one currently being planned.

## 6. Conclusions

Integrating GL and beta-glucan into the aggregated fuzzy evaluation yields a coherent, food-based dietary circle oriented toward T2D prevention that can serve as a reference for counseling, menu planning, and intervention studies. Our results are not the optimum but a step in the right direction. The optimization was stopped as soon as the recommendations for all nutrients considered were met (evaluation > 0.9) and is therefore comparable with the procedure used in [26]; further optimization steps toward the optimum lead to changes far from common eating behavior. Whether the derived diet actually reduces the risk of T2D remains to be tested in intervention studies. To incorporate GL and beta-glucan into general nutritional counseling for T2D prevention, it would be highly desirable to include both in the BLS (German Nutrient Database).

## Figures and Tables

**Figure 1:**
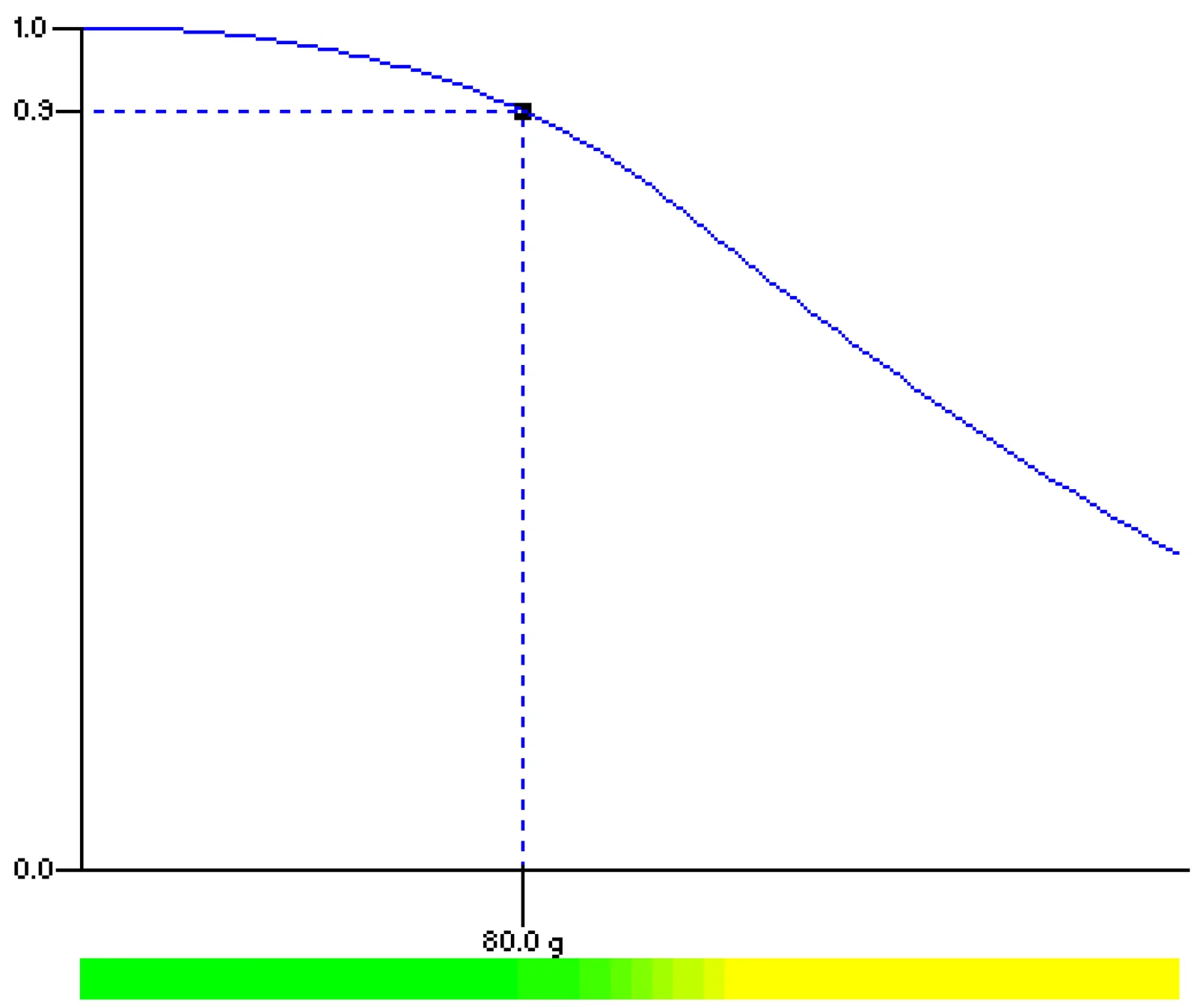
Fuzzy set (membership function) for the evaluation of daily glycemic load (GL) intake, referenced to an energy intake of 2000 kcal/d. Intakes up to 80 g/d are evaluated with at least 0.9 (desirable); above this value, the evaluation decreases continuously, in accordance with the DDG classification of “moderate” (80–160 g/d) and “concerningly high” (>160 g/d) GL [21]. The colored bar indicates the evaluation range (green: good; yellow: less favorable).

**Figure 2:**
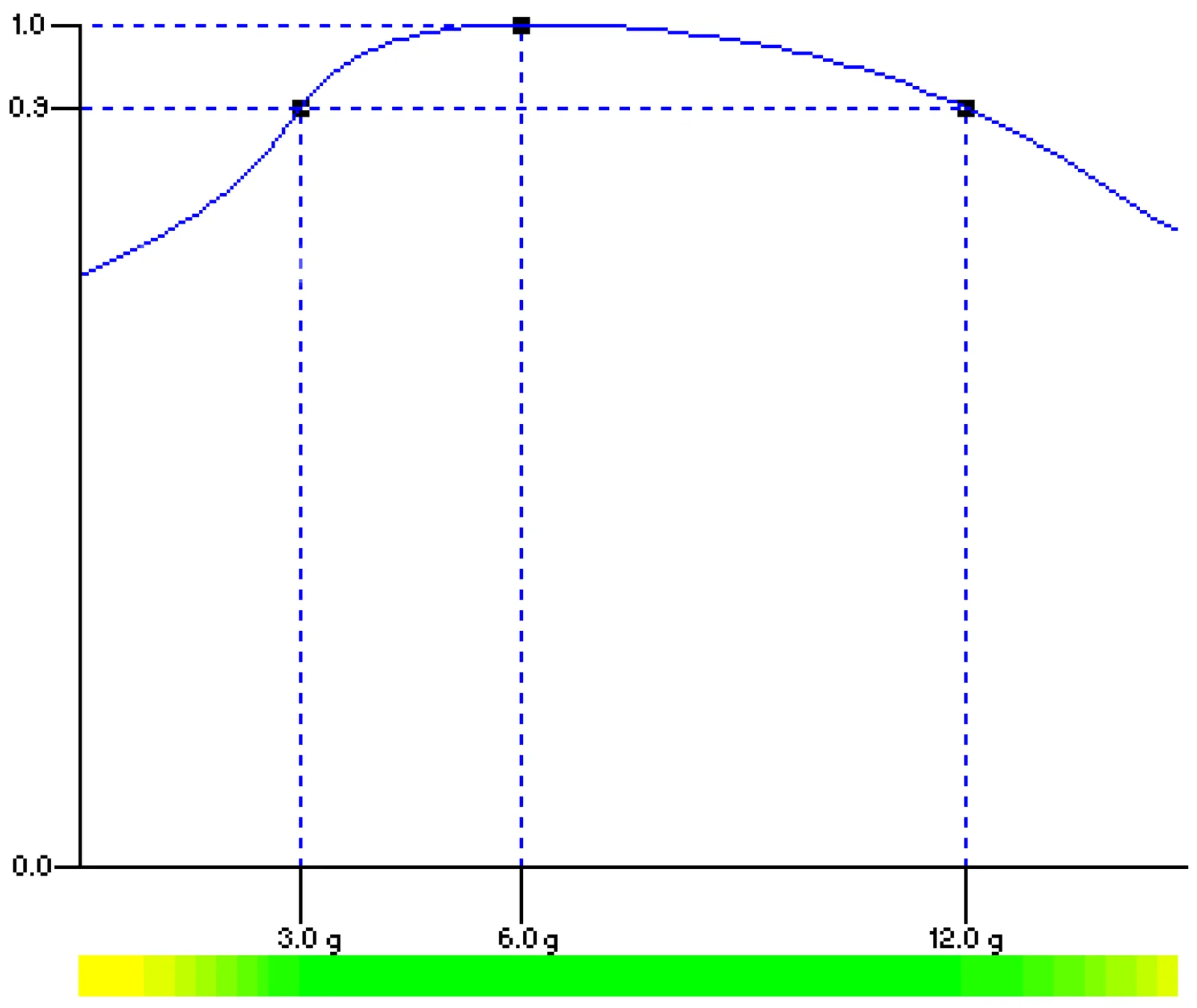
Fuzzy set (membership function) for the evaluation of daily beta-glucan intake in adults. The evaluation reaches 0.9 at 3 g/d (lower limit of the desirable range), the optimum (1.0) at 6 g/d, and 0.9 again at 12 g/d (upper limit of the desirable range); at higher intakes, it declines gradually. The colored bar indicates the evaluation range (green: good; yellow: less favorable).

**Figure 3:**
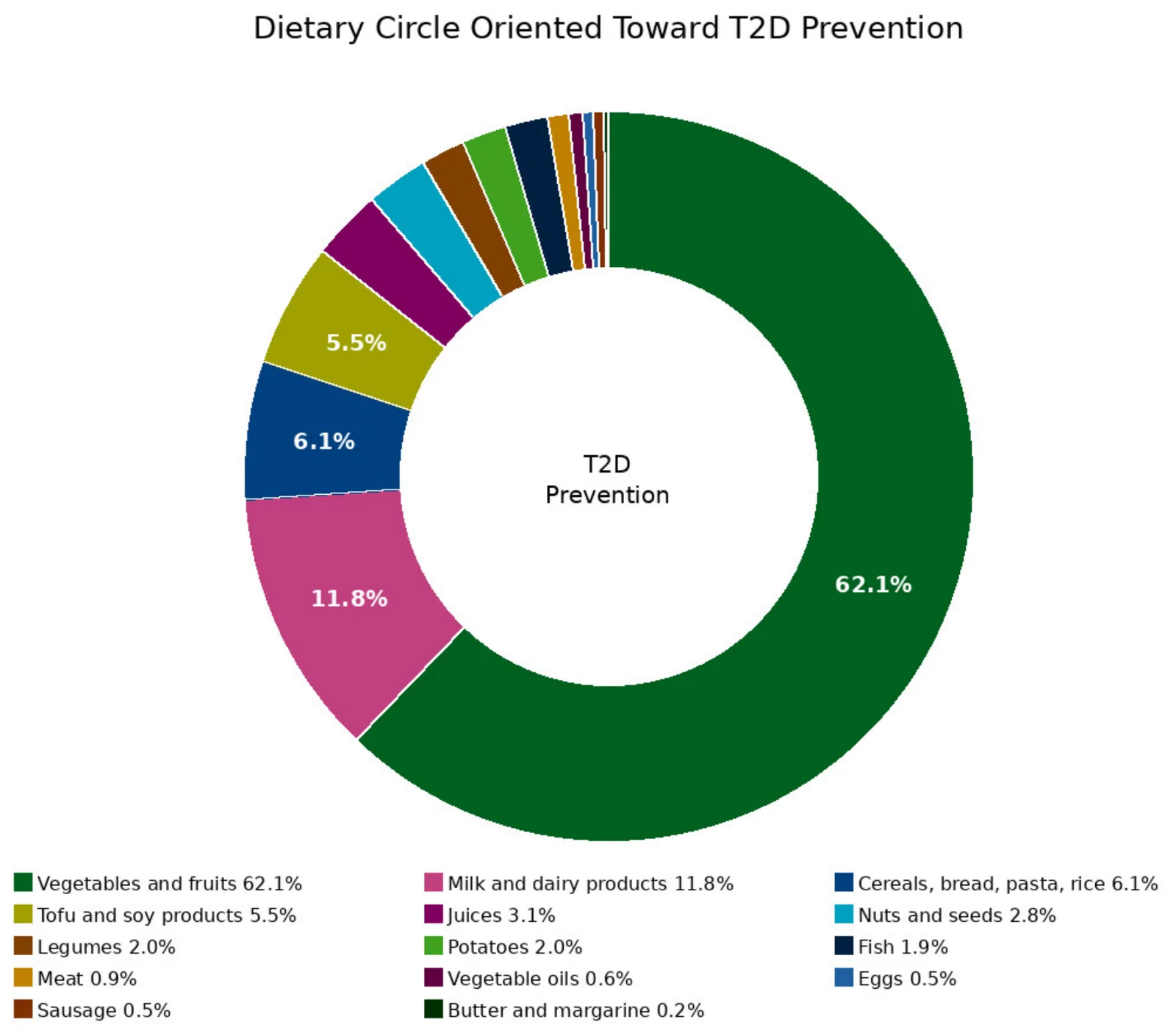
Dietary circle oriented toward T2D prevention, derived by fuzzy optimization (Table 1, third column). Segment areas are proportional to the weekly intake quantities (g/week) of the food groups; percentages indicate each group’s share of the total food weight (beverages not included).

**Figure 4:**
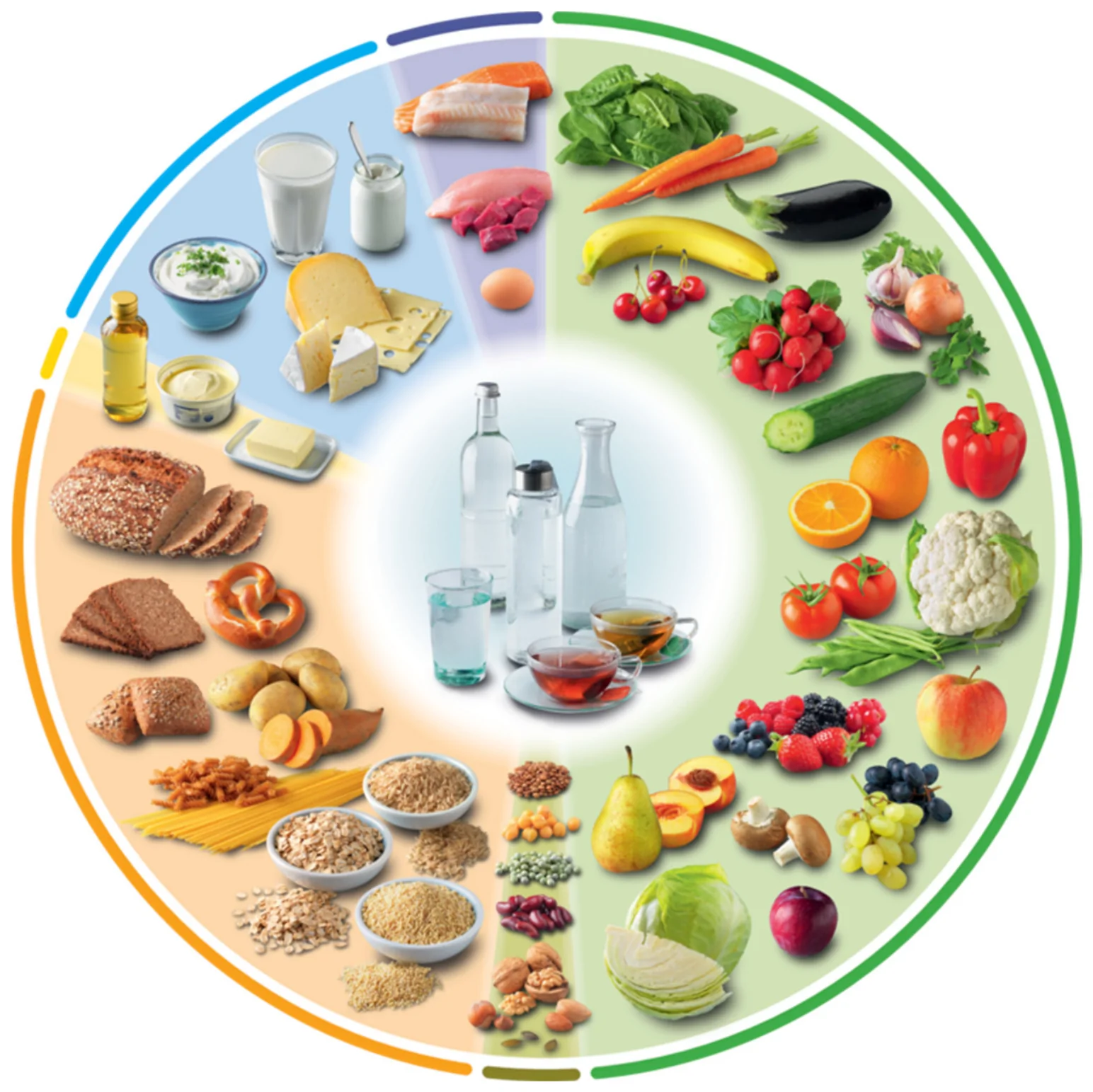
Classical food circle of the German Nutrition Society (DGE) [29]. Unlike in Figure 3, segment sizes reflect portion sizes and frequencies rather than food weights.

**Table 1:** Weekly intake quantities (g/week) per food group for omnivorous adults aged 18–65 years: optimized quantities according to [26] (Scenario 1, Variant 3; Table e2 in [28]) and their adaptation by fuzzy optimization incorporating the evaluation of GL and beta-glucan and one additional food group (tofu and soy products). The last column shows the contribution of each food group to daily GL and beta-glucan intake (g/d). ↑ increase, ↓ decrease (bold arrows: marked change), = nearly unchanged.

Food Group	Intake (g/Week) Scenario 1, Variant 3 [26]	Intake After Fuzzy Optimization (g/Week)	Contribution to Glycemic Load/Beta-Glucan (g/d)
Vegetables, Lettuce and Fruits	3829	7894 **↑**	27.6/0.0
Juices	427	400 =	3.8/0.0
Vegetable Oils	84	80 =	0.0/0.0
Legumes	35	250 **↑**	3.2/0.0
Nuts and Seeds	217	350 ↑	2.0/0.0
Potatoes	259	250 =	3.6/0.0
Cereals, Bread, Pasta, Rice—at least 1/3 Whole Grain	2233	780 **↓**	32.1/3.1
Milk and Dairy Products	2653	1500 **↓**	4.3/0.0
Eggs	84	60 =	0.0/0.0
Fish	182	240 ↑	0.0/0.0
Meat (Beef, Pork, Poultry) & Sausage	63 56	120 ↑ 60 =	0.0/0.0 0.0/0.0
Butter and Margarine	77	20 **↓**	0.0/0.0
Tofu and Soy Products without Sugar	-	700 **↑**	0.3/0.0

**Table 2:** Daily nutrient intake after fuzzy optimization, averaged recommendation (mean of the reference values for women and men [6,26]), and fuzzy evaluation of each intake (scale 0–1; ≥0.9 corresponds to compliance with the recommendation). Vitamin D, fluoride, copper, and manganese were not considered (see text). Aggregated fuzzy evaluation: 0.894.

Nutrient	Averaged Recommendation	Intake	Evaluation
Energy	1930 kcal	1630 kcal	0.91
Fat, E%	<40%	34%	0.97
Carbohydrates E%	-	40%	0.98
Protein	52 g	80.8 g	1.0
Water	2.12 L	3.2 L	1.0
Retinol equivalent	776 µg	4.7 mg	0.98
Vitamin E	13 mg	23.7 mg	1.0
Vitamin K	68 µg	750 µg	0.99
Vitamin B1	1.1 mg	1.6 mg	1.0
Vitamin B2	1.2 mg	1.54 mg	1.0
Niacin equivalent	13 mg	39.9 mg	0.95
Pantothenic acid	5 mg	5.5 mg	1.0
Vitamin B6	1.5 mg	2.36 mg	1.0
Biotin	40 µg	87.1 µg	1.0
Folic acid	0.3 mg	0.54 mg	0.99
Vitamin B12	4 µg	2.9 µg	0.99
Vitamin C	103 mg	206 mg	1.0
Sodium	1.5 g	2.1 g	1.0
Potassium	4 g	4.9 g	1.0
Calcium	1 g	0.97 g	1.0
Magnesium	325 mg	542 mg	0.96
Phosphorus	700 mg	1360 mg	0.99
Iron	15 mg	16.5 mg	1.0
Zinc	9 mg	10.2 mg	0.99
Dietary fiber	30 g	40.6 g	1.0
Sucrose, E%	<10%	6.7%	0.96
Cholesterol	<0.3 g	0.1 g	0.99
Alcohol	<10 g	0 g	1
Saturated fatty acids, E%	<10%	9.6%	0.91
*n*-3 fatty acids, E%	0.5%	1.0%	0.99
*n*-6 fatty acids, E%	2%	7.0%	0.94
Glycemic load	<80 g	77.0 g	0.91
Beta glucan	>3 g	3.2 g	0.92

## Data Availability

All data supporting the findings of this study are contained within the article (Table 1 and Table 2). Further details are available from the corresponding author on request.
